# Supplementary material for: Rapid evolution of bacterial mutualism in the plant rhizosphere
Source: Nat Commun. 2021 Jun 22;12:3829. doi: 10.1038/s41467-021-24005-y (PMC8219802; doi:10.1038/s41467-021-24005-y)
Supplement: Supplementary file 1 — Supplementary Information [file 41467_2021_24005_MOESM1_ESM.pdf]

Supplementary Information for

Rapid evolution of bacterial mutualism in the plant rhizosphere

The following file includes:

Figs. S1 to S10

Tables S1 to S4

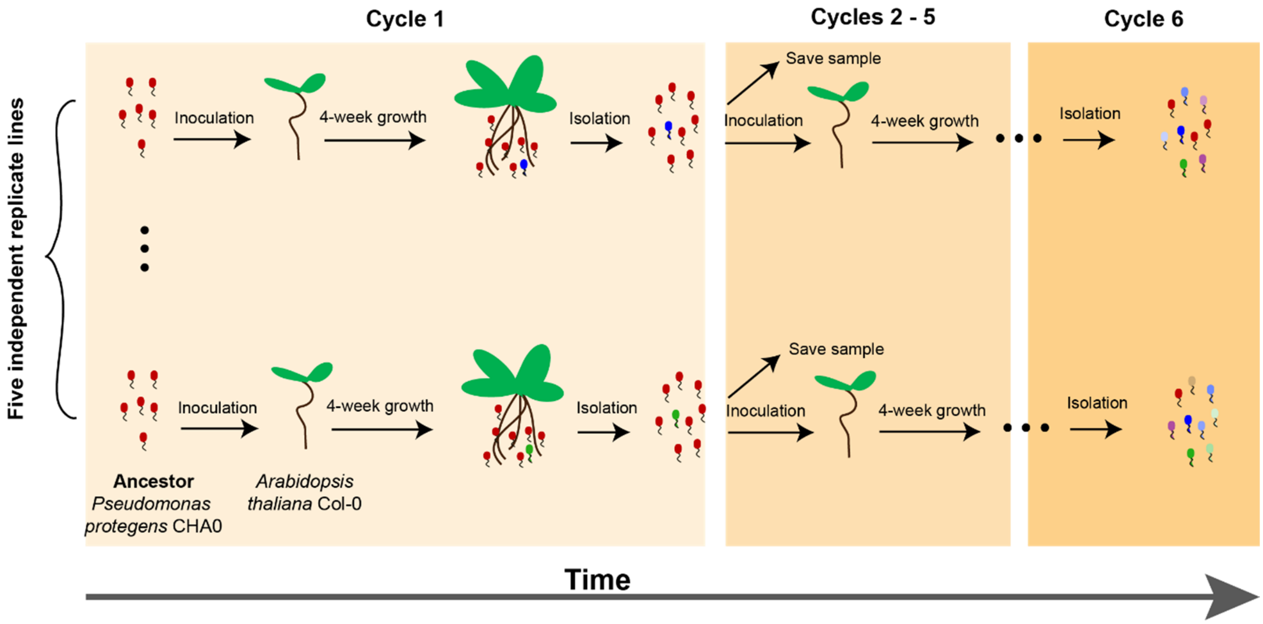

Figure S1. Overview of the experimental design. In this study, we experimentally evolved *Pseudomonas protegens* CHA0 in the rhizosphere of sterile *Arabidopsis thaliana* plants**.** We used a gnotobiotic, organic carbon-free soil system in which bacterial fitness strictly depended on their interaction with plants. We set up five independent plant replicate lines, which were passed over six plant growth cycles (4 weeks each). To this end, 10^6 cells of the ancestral *P. protegens* CHA0 strain were introduced to the rhizosphere of two *A. thaliana* seedlings grown in sterile silver sand supplemented with a plant nutrient solution in sterile ECO2 boxes. At the end of each growth cycle, the rhizosphere bacterial population was harvested, and 10^6 cells were inoculated onto a new plant. The remaining bacteria were kept as frozen stock at -80 °C. At the end of the experiment, bacteria from the -80 °C stock were plated on 3 g l^{-1} Tryptic Soy Agar and sixteen single bacterial isolates were randomly selected from each replicate line at the end of the second, fourth and sixth growth cycle. In total, 240 evolved isolates and sixteen ancestral isolates were phenotyped regarding traits associated with bacterial fitness and mutualistic activity with the plant.

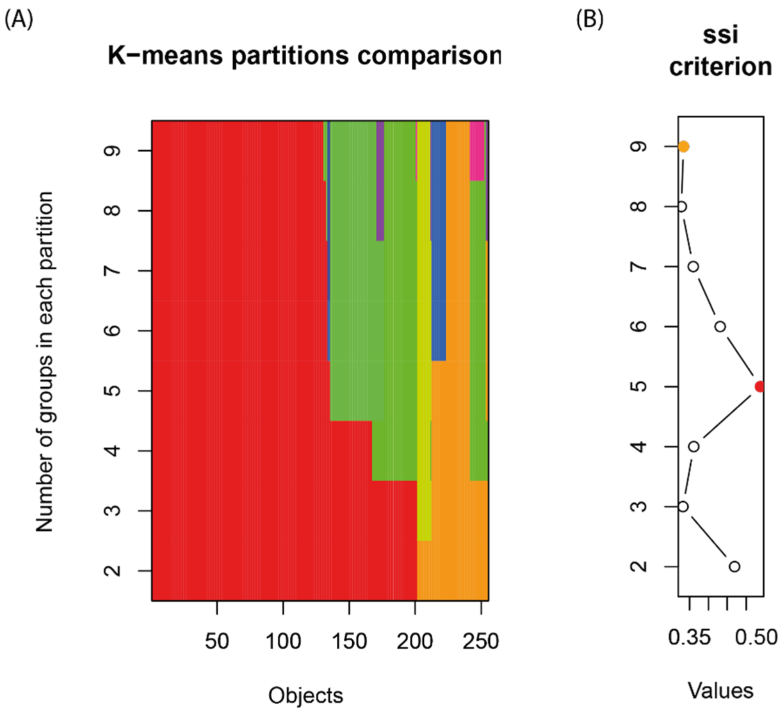

Figure S2. K-means clustering analysis of evolved *Pseudomonas protegens* CHA0 isolates. Isolated colonies were classified based on 14 phenotypic traits associated with bacterial fitness in the rhizosphere and mutualistic activity with the plant. In panel A, the x-axis ("Objects") represents the 256 screened isolates while the y-axis represents the potential number of clusters (K) shown in different colours. Panel B shows the SSI criterion values indicating the most parsimonious number of clusters needed to classify isolates into distinct phenotypic groups. Based on this analysis, we classified the isolates into five clusters (the highest SSI criterion value).

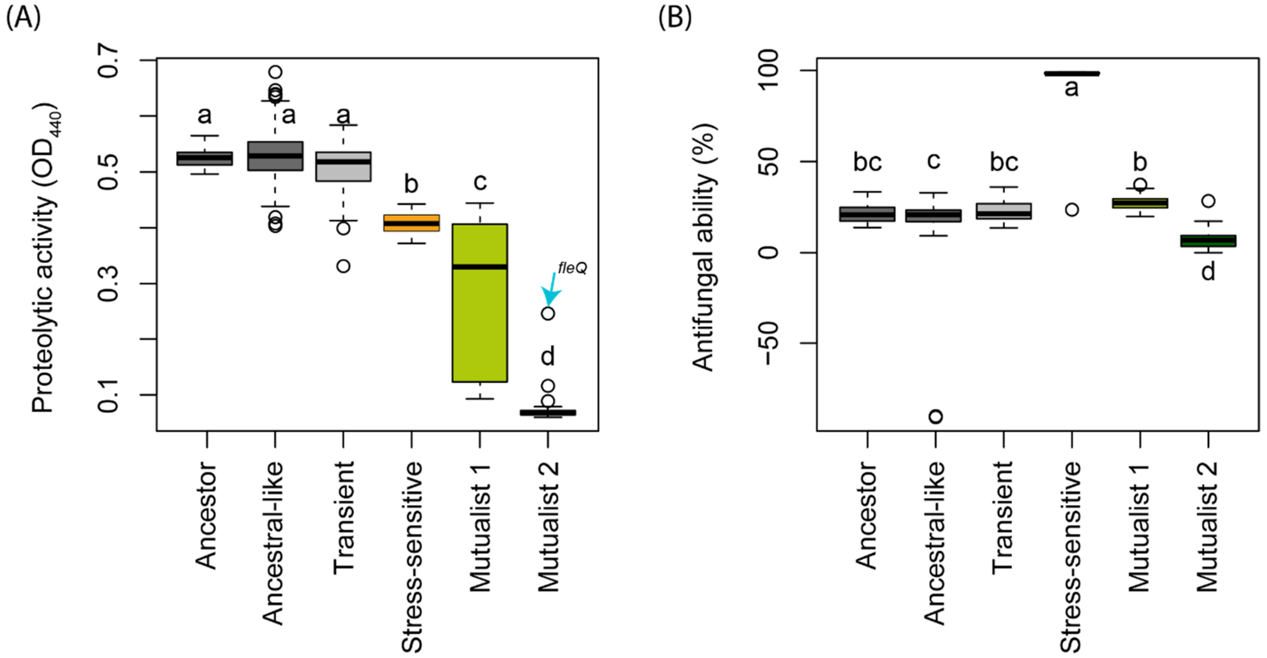

Figure S3. Comparing the differences in extracellular proteolytic and antifungal activity of ancestral and evolved *Pseudomonas protegens* CHA0 isolates. We measured the proteolytic (A) and antifungal activity (B) as a proxy for secondary metabolite production. In total, we characterized 256 isolates including ‘ancestral’ (n=16), ‘Ancestral-like’ (n=119), ‘Transient’ (n=41), ‘Stress-sensitive’ (n=11), ‘Mutualist 1’ (n=37) and ‘Mutualist 2’ (n=31) (Supplementary data 1). In panel A, the blue arrow indicates one isolate that was phenotypically clustered as mutualist 2, but genetically bearing a unique *fleQ* mutation. In all panels, bacterial phenotype groups are displayed on different colours (black: ancestor; dark grey: ancestral-like; light grey: transient; orange: stress-sensitive, light green: mutualist 1 and dark green: mutualist 2). All boxplots show median (center line), interquartile range (25%-75%), and whiskers that extended 1.5 times the interquartile range. Statistical testing was carried out using one-way ANOVA. Different letters indicate significant differences based on a Tukey HSD test (α=0.05). Data for all panels are provided in the Source Data file.

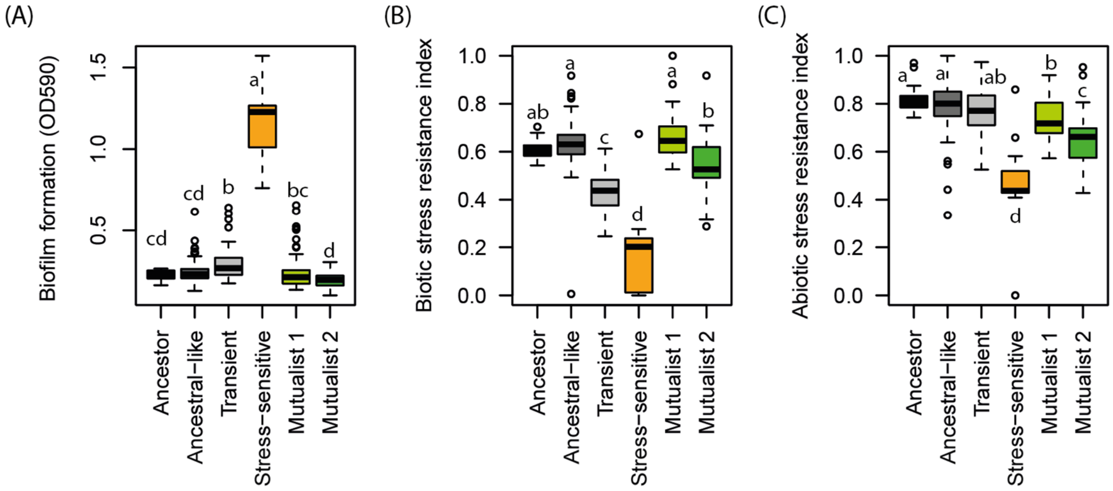

Figure S4. Comparing biofilm formation, biotic and abiotic stress resistance of ancestral and evolved *Pseudomonas protegens* CHA0 isolates. In total, we characterized 256 isolates including ‘ancestral’ (n=16), ‘Ancestral-like’ (n=119), ‘Transient’ (n=41), ‘Stress-sensitive’ (n=11), ‘Mutualist 1’ (n=37) and ‘Mutualist 2’ (n=31); Supplementary data 1. Panels A, B and C show biofilm formation, biotic stress resistance index (normalised PC1 of combined ability to grow in the presence of sub lethal doses of the antibiotics streptomycin, tetracycline, and penicillin) and abiotic stress resistance index (normalised first principal component of combined ability of each isolate to grow under oxidative stress, water potential stress and salt stress), respectively. In all panels, bacterial phenotype groups are displayed on different colours (black: ancestor; dark grey: ancestral-like; light grey: transient; orange: stress-sensitive, light green: mutualist 1 and dark green: mutualist 2). All boxplots show median (center line), interquartile range (25%-75%), and whiskers that extended 1.5 times the interquartile range. Statistical testing was carried out using one-way ANOVA. Different letters indicate significant differences based on a Tukey HSD test (α=0.05). Data for all panels are provided in the Source Data file.

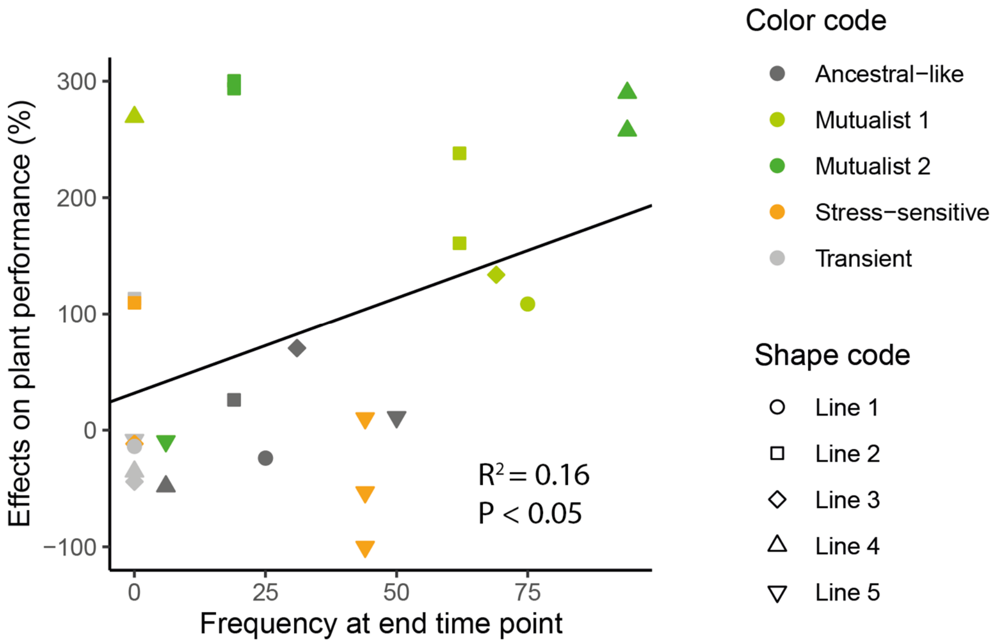

Figure S5. Positive relationship between phenotype frequency at the end of the selection experiment and isolate effect on the plant performance. Five representative bacterial isolates from each phenotype in addition to the ancestor were selected to measure their effects on *Arabidopsis thaliana* growth in terms of combined 'Plant performance' index (30 isolates altogether, each replicate line represented (n=5); See Table S2). The y-axis represents the beneficial effect of isolates on plant performance relative to the ancestor. Values on the x-axis show the relative abundance of evolved phenotypes in their respective selection lines at the end of the sixth plant growth cycle (see Figure 2A). Bacterial phenotype groups are displayed on different colours (black: ancestor; dark grey: ancestral-like; light grey: transient; orange: stress-sensitive, light green: mutualist 1 and dark green: mutualist 2) and shapes represent different phenotypes and selection lines, respectively (line1: circle; line2: square; line3: diamond; line4: triangle and line5: top-down triangle). Black line shows linear regression (R^2=0.16, P= 0.048). Data for the panel is provided in the Source Data file.

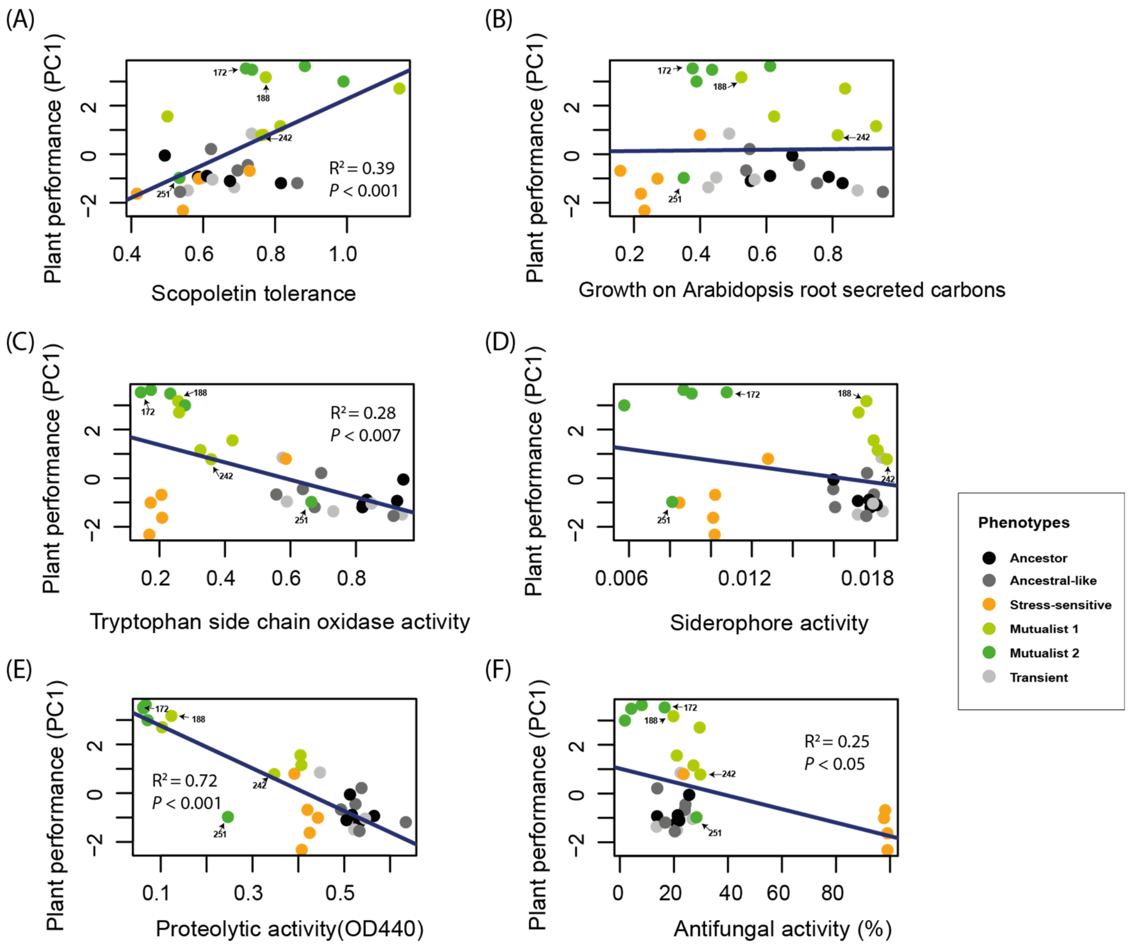

Figure S6. Associations between plant performance and different bacterial life-history traits. Ancestor and five representative bacterial isolates from each phenotype group were characterised regarding their effects on *Arabidopsis thaliana* growth in terms of combined 'Plant performance' index (30 isolates altogether, each replicate line represented (n=5); See Table S2; y-axes). Values on the x-axes of panels A-F show variation regarding scopoletin tolerance, growth on *Arabidopsis* root secreted carbons, tryptophan side chain oxidase activity, siderophore activity, proteolytic activity, and antifungal activity, respectively. The sample IDs of four isolates from the two mutualistic phenotype groups are highlighted on labels (See Table S2) in all panels. In all panels, bacterial phenotype groups are displayed on different colours (black: ancestor; dark grey: ancestral-like; light grey: transient; orange: stress-sensitive, light green: mutualist 1 and dark green: mutualist 2). All boxplots show median (center line), interquartile range (25%-75%), and whiskers that extended 1.5 times the interquartile range. Black lines show linear regression. Data for all panels are provided in the Source Data file.

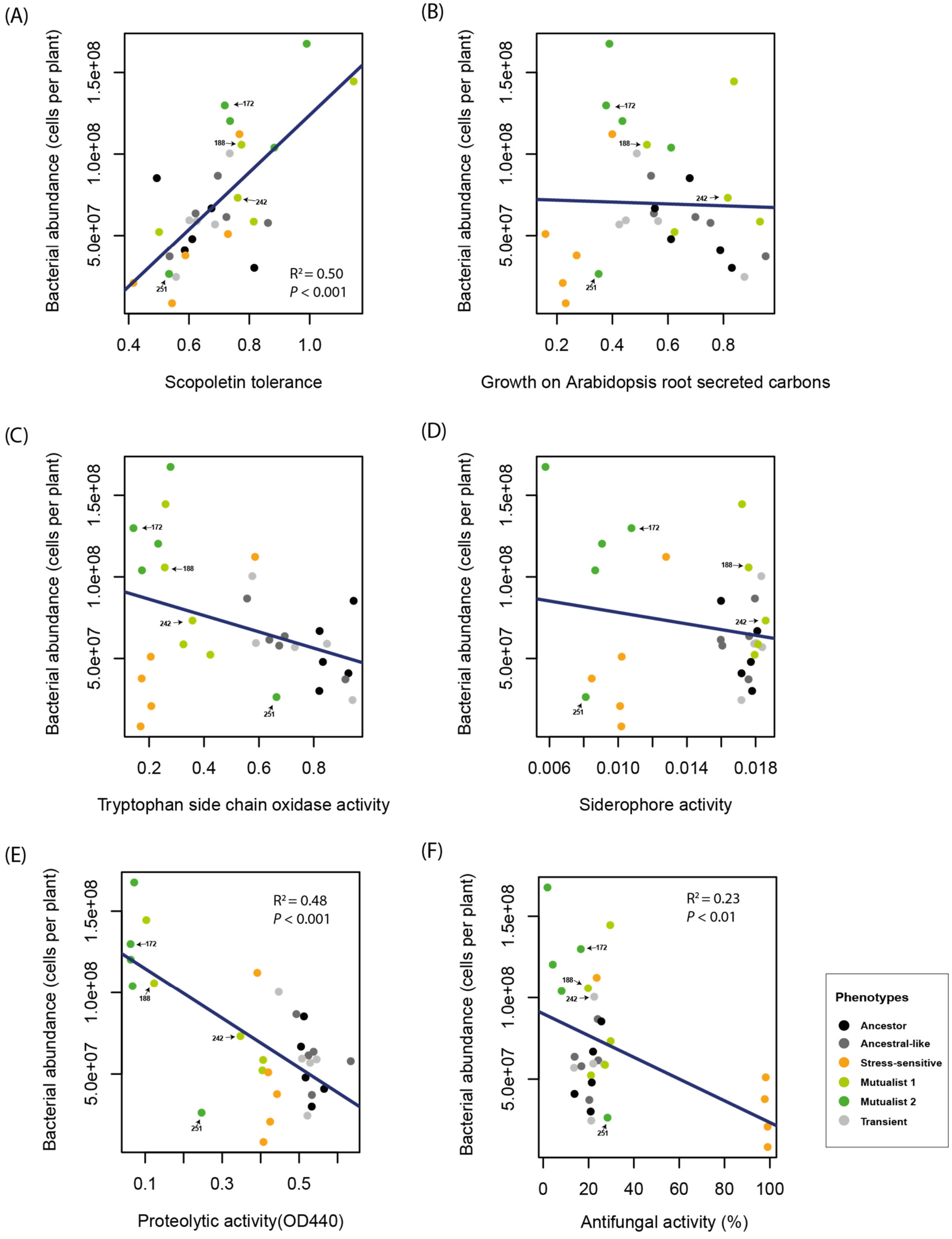

Figure S7. Associations between bacterial abundance and different bacterial life-history traits. Ancestor and five representative bacterial isolates from each phenotype group were characterised regarding their growth on *Arabidopsis thaliana* roots (30 isolates altogether, each replicate line represented (n=5); See Table S2; y-axes). Values on the x-axes of panels A-F show

variation regarding scopoletin tolerance, growth on *Arabidopsis* root secreted carbons, tryptophan side chain oxidase activity, siderophore activity, proteolytic activity, and antifungal activity, respectively. The sample IDs of four isolates from the two mutualistic phenotype groups are highlighted on labels (See Table S2) in all panels. In all panels, bacterial phenotype groups are displayed on different colours (black: ancestor; dark grey: ancestral-like; light grey: transient; orange: stress-sensitive, light green: mutualist 1 and dark green: mutualist 2). All boxplots show median (center line), interquartile range (25%-75%), and whiskers that extended 1.5 times the interquartile range. Black lines show linear regressions. Data for all panels are provided in the Source Data file.

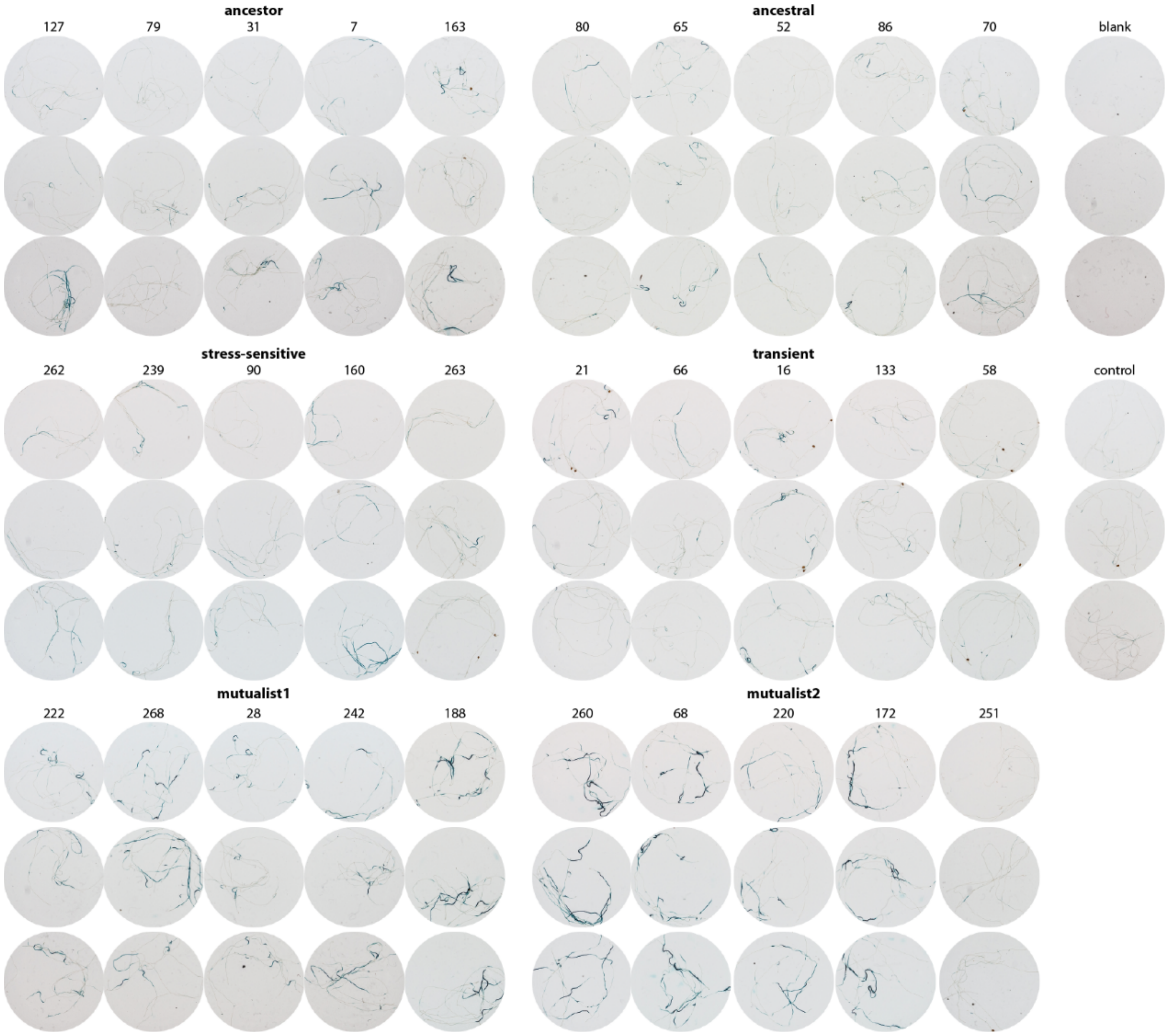

Figure S8. Induction of *MYB72* assayed in a GUS reporter line in *Arabidopsis thaliana* by the ancestor and a subset of evolved *Pseudomonas protegens* CHA0 isolates. GUS staining was performed at 2 days after bacterial inoculation (n = 3 biological plant replicates, each containing 5 to 6 seedlings). Bacteria- and control-treated, GUS-stained roots are grouped by bacterial phenotype and for each isolate the respective Sample ID is shown on top of the three biological replicates. See Table S2 for detailed information of the isolates. Data are provided in the Source Data file.

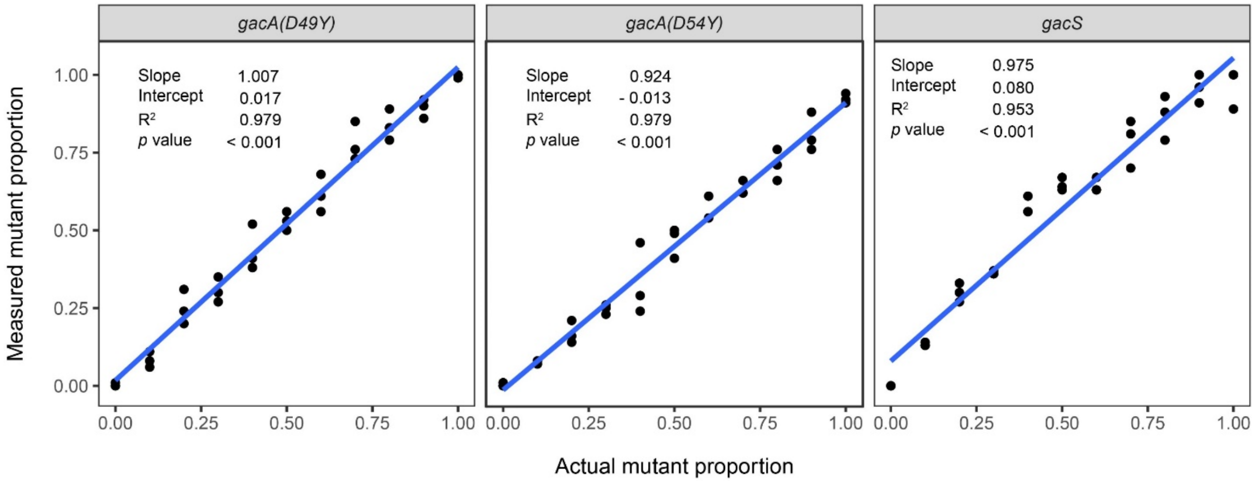

Figure S9 Standard curves of measured mutant versus ancestor proportion as a function of the actual proportion, using series of mixed samples with known proportions (0%, 10%, 20%, 30%, 40%, 50%, 60%, 70%, 80%, 90% and 100% of mutant templates). Relative densities of mutants *gacA*^{D49Y}, *gacA*^{D54Y}, and *gacS*^{G27D} were measured by PCR-based high-resolution melting profile (RQ-HRM) analysis (n=3). In each plot, the black dots represent the measurement replicates and blue line show linear regression. Data for all panels are provided in the Source Data file.

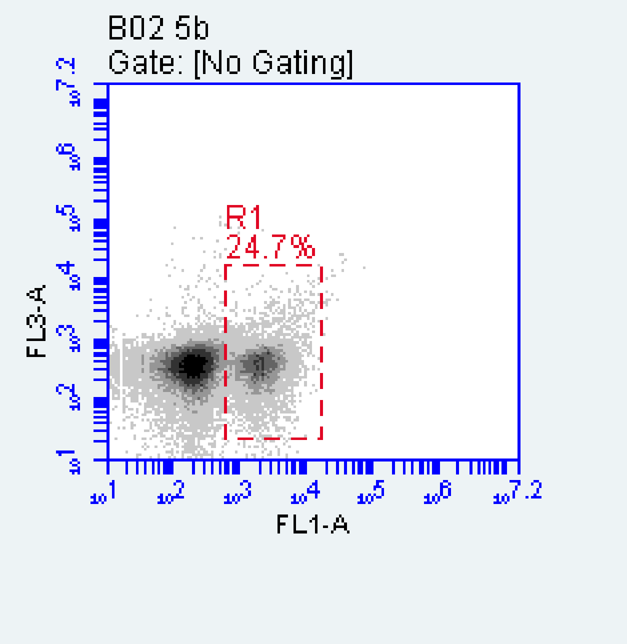

Plot 8: B02 5b	Count	Events / µL	% of This Plot	% of All
All	35,490	3549	100.00%	100.00%
R1	8,774	877	24.72%	24.72%

Figure S10. Flow cytometry was used to count GFP-labelled *Pseudomonas protegens* CHA0 cells with thresholds for Forward Scatter (FSC) and Side Scatter (SSC) set to 2000 and 8000, respectively. The example sample name "B02 5b" shown here denotes a generation 2, replicate 5 sample. Gating (FL1-A) is used to distinguish the bacterial GFP signal from the noise: R1 region shows the targeted GFP-labelled *P. protegens* CHA0 cells – everything outside this area is considered as noise. The measured samples were root suspensions that were mixed in buffer solution (10 mM $MgSO_4$) and diluted for 20 times (10 µl into 190 µl). The table below, provided by the software, indicates that the concentration of the original cell suspension of this example sample was 876 events (cells) times 20. The data are analysed with BD Accuri™ C6 Plus Analysis Software.

Table S1. *In vitro* measurement of different aspects of bacterial life-history traits, including bacterial growth, tolerance to diverse abiotic and biotic stresses, production (or activity) of bioactive compounds and antimicrobial activity. In total, 14 different phenotypic traits were measured for 256 *Pseudomonas protegens* CHA0 isolates in this study including 16 isolates of the ancestor.

**Aspects of life-history traits**	**Details of measured traits**
Bacterial growth yield	Bacterial growth yield in King's medium B
Stress tolerance	Growth yield under abiotic stresses: oxidative stress, water potential stress, salt stress<br><br>Growth yield under biotic stresses (antibiotics): streptomycin stress, tetracycline stress, penicillin stress
Production (or activity) of bioactive compounds	IAA (auxin) production, siderophore activity, quorum sensing (TSO) activity, proteolytic activity, biofilm formation
Antimicrobial activity	Antifungal activity: *Verticillium dahliae*<br><br>Antibacterial activity: *Ralstonia solanacearum*

Table S2. Description of five ancestral and 25 evolved *Pseudomonas protegens* CHA0 isolates, which were included in phenotyping and genotyping assays. "Sample ID" is the unique identifier of each isolate and five isolates were selected from each phenotype class. The "Plant cycle" column refers to the plant growth cycle from which the isolate was collected (see Figure S1) and the "Replicate" column refers to the independent plant replicate selection line. Mutated genes were identified using whole genome re-sequencing. On average, each evolved isolate encompassed 2-3 mutations relative to the ancestral sequence that are typically non-synonymous in nature, *i.e.* they directly affect predicted protein sequence and/or protein length. Matching Sample ID superscript numbers shown in bold (133 and 242, 66 and 222, 28 and 220) indicate paired samples used in relative competition assays presented in Figure 5.

**Phenotype**	**Sample ID**	**Plant cycle**	**Replicate**	**Genotype[1]**
Ancestor	7	0	Ancestor	_
Ancestor	31	0	Ancestor	_
Ancestor	79	0	Ancestor	_
Ancestor	127	0	Ancestor	_
Ancestor	163	0	Ancestor	_
Ancestral-like	52	4	Line 2	*galE*^{V32M}
Ancestral-like	65	2	Line 3	_
Ancestral-like	70	4	Line 5	_
Ancestral-like	80	4	Line 4	_
Ancestral-like	86	4	Line 1	*oafA*^{Y335X} · *RS17350*$^{A77A.fsX14}$ · *wbpM*^{G79R}
Transient	16	4	Line 2	*galE*^{V32M} · *accC*^{E413K}
Transient	21	2	Line 4	*hult*$^{\underline{786C>T}}$
Transient	58	4	Line 5	_
Transient	**66[3]**	4	Line 3	*oafA*$^{K338S.fsX18}$
Transient	**133[1]**	2	Line 1	*oafA*^{Y335X} · *RS17350*$^{A77A.fsX14}$
Stress-sensitive	90	4	Line 3	*nlpD*^{Q197P}
Stress-sensitive	160	4	Line 2	*galE*^{V32M} · *accC*^{E413K} · *yvaQ2*$^{-9G>T}$
Stress-sensitive	239	6	Line 5	*rpoS*^{Q65X} · *tetR*^{Y127X}

Stress-sensitive	262	6	Line 5	$\mathit{rpoS}^{\mathrm{Q65X}}$
Stress-sensitive	263	6	Line 5	$\mathit{rpoS}^{\mathrm{Q65X}}$
Mutualist 1	**28**[2]	4	Line 2	$\mathit{galE}^{\mathrm{V32M}} \cdot \mathit{accC}^{\mathrm{E413K}}$
Mutualist 1	188	4	Line 4	$\mathit{gacA}^{\underline{\mathrm{-40T>A}}}$
Mutualist 1	**222**[3]	6	Line 3	$\mathit{oafA}^{\mathrm{K338S.fsX18}} \cdot \mathit{gacS}^{\mathrm{G27D}}$
Mutualist 1	**242**[1]	6	Line 1	$\mathit{oafA}^{\mathrm{Y335X}} \cdot \mathit{RS17350}^{\mathrm{A77A.fsX14}} \cdot \mathit{gacA}^{\mathrm{D49Y}}$
Mutualist 1	268	6	Line 2	$\mathit{galE}^{\mathrm{V32M}} \cdot \mathit{accC}^{\mathrm{E413K}} \cdot \mathit{gacA}^{\mathrm{G97S}} \cdot \mathit{mraZ}^{\underline{\mathrm{-211A>G}}}$
Mutualist 2	68	4	Line 4	$\mathit{gacA}^{\underline{\mathrm{-40T>A}}} \cdot \mathit{RS11820}^{\underline{\mathrm{33C>T}}}$
Mutualist 2	172	4	Line 2	$\mathit{gacA}^{\mathrm{Y183S}}$
Mutualist 2	**220**[2]	6	Line 2	$\mathit{galE}^{\mathrm{V32M}} \cdot \mathit{accC}^{\mathrm{E413K}} \cdot \mathit{gacA}^{\mathrm{D54Y}}$
Mutualist 2	251	6	Line 5	$\mathit{fleQ}^{\mathrm{R320Q}}$
Mutualist 2	260	6	Line 4	$\mathit{gacA}^{\mathrm{E38X}} \cdot \mathit{RS11785}^{\mathrm{S256C}} \cdot \mathit{flhA}^{\mathrm{H393Q.fsX15}}$

[1]X represents a stop codon (at its relative position in case of a shifted frame); fs, frame shift; sequence change positions are relative to the cDNA. For example, $\mathit{flhA}^{\mathrm{H393Q.fsX15}}$ means this mutation (a single deletion) lead to an amino acid change to Q from H at position 393 due to the frame shift (fs) caused by the deletion followed by a stop codon X after another 15 amino acids (X15). Notably, these additional amino acids are in a different frame and thus represent a totally different sequence that the wild type allele. Synonymous mutations, or mutations that are found in the intergenic region and listed with a preceding minus symbol, are depicted on the nucleotide level relative to the cDNA and are underlined.

Table S3. Overview of the affected genes that are identified in the 25 evolved *Pseudomonas protegens* CHA0 isolates (See Table S2).

Gene	*Product*	*Locus tag*	*DNA change*	*Effect*[1]
accC	Biotin carboxylase, acetyl-CoA carboxylase	PFLCHA0_RS03400	c.1237 G>A	p.E413K
flhA	Flagellar biosynthesis protein FlhA	PFLCHA0_RS08490	c.1154 T deleted	p.H393Q.fsX15
gacA	Response regulator GacA	PFLCHA0_RS17965	c.548 A>C	p.Y183S
gacA	Response regulator GacA	PFLCHA0_RS17965	c.289 G>A	p.G97S
gacA	Response regulator GacA	PFLCHA0_RS17965	c.160 G>T	p.D54Y
gacA	Response regulator GacA	PFLCHA0_RS17965	c.145 G>T	p.D49Y
gacA	Response regulator GacA	PFLCHA0_RS17965	c.112 G>T	p.E38X
gacA	Response regulator GacA	PFLCHA0_RS17965	-40 T>A	promoter
galE	UDP-glucose 4-epimerase	PFLCHA0_RS09920	c.94 G>A	p.V32M
RS11785	LysR family transcriptional regulator	PFLCHA0_RS11785	c.766 A>T	p.S256C
hutI	Imidazolonepropionase	PFLCHA0_RS02080	c.786 C>T	synonymous
RS11820	PaaI family thioesterase	PFLCHA0_RS11820	c.33 C>T	synonymous
yvaQ2	Metyl-accepting chemotaxis protein	PFLCHA0_RS13000	-9bp G>T	promoter
mraZ	Transcriptional regulator mraZ	PFLCHA0_RS25175	-211bp A>G	promoter
nlpD	Lipoprotein nlpD/lppB/LysM domain-containing protein	PFLCHA0_RS31060	c.590 A>C	p.Q197P
fleQ	Sigma-54-dependent Fis family transcriptional regulator	PFLCHA0_RS08340	c.959 G>A	p.R320Q
oafA	O-acetyltransferase OafA	PFLCHA0_RS09890	c.1005 C>A	p.Y335X
oafA	O-acetyltransferase OafA	PFLCHA0_RS09890	c.1009 A deleted	p.K338S.fsX18

wbpM	Polysaccharide biosynthesis protein/NDP-sugar epimerase	PFLCHA0_RS21855	c.235 G>C	p.G79R
rpoS	RNA polymerase sigma factor RpoS	PFLCHA0_RS06125	c.193 C>T	p.Q65X
RS17350	Methyltransferase domain-containing protein	PFLCHA0_RS17350	c.116 C deleted	p.A77A.fsX14

[1]Mutations that are identified in the intergenic region are identified by the adjacent, downstream gene and labeled with *promoter.* X, represents a stop codon (at its relative position in case of a shifted frame); fs, frame shift; sequence change positions are relative to the cDNA (see Table S2 for detailed explanation).

Table S4 Primers and probes used for high-resolution melting (HRM) analysis. For the two *gacA* mutants the same set of primers was used. Underlined bases indicate the position of the single nucleotide point (SNP) mutations within the probe sequences. ΔTm (°C) indicates the melting temperature difference between WT-probe duplex and mutant-probe duplex

Target gene	Strain ID	SNP locus	Forward primer (excess)	Reverse primer (limiting)	Amplicon size	Probe sequence	Probe length	Target strand	Perfect match/ mismatch	ΔTm (°C)
gacA^{D49Y}	242	145G>T	5'-ATCGATGGCCTGCAAGTAGT-3’	5'-CGGGTAGGAAAGGGATCTTC-3’	206 bp	5’-CATCAGGACCACATCGGGCTTCAGCTCCCG-/C3/3’	30nt	WT	G::C / T::C	5.31
gacA^{D54Y}	220	160G>T				5’-TGGCATCTTGACGTCCATCAGGACCACATC-/C3/3’	30nt	WT	G::C / T::C	4.92
gacS^{G27D}	222	80G>A	5’-GCGTACTGTTGCTGACCTTG-3’	5’-AGCATCTGGGTGTTGTGGTT-3’	178bp	5’-AGGTGAAGTAGCCGCCCAGCACCAAAGCCA-/C3/3’	30nt	WT	G::C / A::C	4.83
