# Supplementary material for: Rapid evolution of bacterial mutualism in the plant rhizosphere
Source: Nat Commun. 2021 Jun 22;12:3829. doi: 10.1038/s41467-021-24005-y (PMC8219802; doi:10.1038/s41467-021-24005-y)
Supplement: Supplementary file 3 — Description of Additional Supplementary Files [file 41467_2021_24005_MOESM3_ESM.pdf]

Description of Additional Supplementary Files

File Name: Supplementary Data 1
Description: **data for 256 selected isolates**. Sheet 1: Overview table of K-means clustering-based phenotype classification of the 256 selected isolates. Sheet 2: Data of fourteen phenotypic traits that were used for K-means clustering. Sheet 3: Carbon use data for the 256 characterised isolates. Sheet 4: PC1 values out of the 14 carbon traits, abiotic stress traits and biotic stress traits. See material and methods for a detailed description of the experimental procedures.

File Name: Supplementary Data 2
Description: **data for 30 selected isolates.** Sheet 1: Summary table for interactions between each of the 30 isolates tested for plant growth, including effect on plant performance and induced root GUS expression. Sheet 2: Recapitulation of the origin (replicate line), time point and phenotype of each of the 30 isolates (3 replicates per isolate) tested in detail for their interactions with the host plant. Sheet 3: Scopoletin sensitivity data. Sheet 4: Relative fitness data: See material and methods for a detailed description of the experimental procedures.

File Name: Supplementary Data 3
Description: **data obtained during the selection experiment.** Sheet 1: Above ground biomass after the first growth cycle. Sheet 2: Frequency dynamics data of each phenotype from each line during the selection experiment.
