# Supplementary material for: Rapid evolution of bacterial mutualism in the plant rhizosphere
Source: Nat Commun. 2021 Jun 22;12:3829. doi: 10.1038/s41467-021-24005-y (PMC8219802; doi:10.1038/s41467-021-24005-y)
Supplement: Supplementary file 7 — Reporting Summary [file 41467_2021_24005_MOESM7_ESM.pdf]

nature research

Corresponding author(s): *Double-blind peer review submissions: write DBPR and your manuscript number here instead of author names.*

Last updated by author(s): *YYYY-MM-DD*

Reporting Summary

Nature Research wishes to improve the reproducibility of the work that we publish. This form provides structure for consistency and transparency in reporting. For further information on Nature Research policies, see our Editorial Policies and the Editorial Policy Checklist.

Statistics

For all statistical analyses, confirm that the following items are present in the figure legend, table legend, main text, or Methods section.

n/a	Confirmed	
☐	☒	The exact sample size (n) for each experimental group/condition, given as a discrete number and unit of measurement
☐	☒	A statement on whether measurements were taken from distinct samples or whether the same sample was measured repeatedly
☐	☒	The statistical test(s) used AND whether they are one- or two-sided<br>*Only common tests should be described solely by name; describe more complex techniques in the Methods section.*
☐	☒	A description of all covariates tested
☐	☒	A description of any assumptions or corrections, such as tests of normality and adjustment for multiple comparisons
☐	☒	A full description of the statistical parameters including central tendency (e.g. means) or other basic estimates (e.g. regression coefficient) AND variation (e.g. standard deviation) or associated estimates of uncertainty (e.g. confidence intervals)
☐	☒	For null hypothesis testing, the test statistic (e.g. F, t, r) with confidence intervals, effect sizes, degrees of freedom and P value noted<br>*Give P values as exact values whenever suitable.*
☒	☐	For Bayesian analysis, information on the choice of priors and Markov chain Monte Carlo settings
☒	☐	For hierarchical and complex designs, identification of the appropriate level for tests and full reporting of outcomes
☒	☐	Estimates of effect sizes (e.g. Cohen's d, Pearson's r), indicating how they were calculated

Our web collection on statistics for biologists contains articles on many of the points above.

Software and code

Policy information about availability of computer code

Data collection	ImageJ (version 1.50i) was used to collect plant performance traits; MATLAB 9.1 was used to quantify the genotype frequencies after competition; BD Accuri c6 plus software was used to collect the bacterial abundance data.
Data analysis	All analyses were carried out under R 3.5 and Python 3.x. Snippy (version 3.2-dev; https://github.com/tseemann/snippy) and BedTools (version 2.27.0) were used to identify and functionally annotate single nucleotide polymorphisms and small insertions and deletions (indels) for each individual strain. PROKKA (version 1.12; https://github.com/tseemann/prokka) was used for full annotation of the updated reference genome. A5 assembly pipeline(version 20160825) was used to determine the CHA-GFP reference genome.

For manuscripts utilizing custom algorithms or software that are central to the research but not yet described in published literature, software must be made available to editors and reviewers. We strongly encourage code deposition in a community repository (e.g. GitHub). See the Nature Research guidelines for submitting code & software for further information.

Data

Policy information about availability of data

All manuscripts must include a data availability statement. This statement should provide the following information, where applicable:

- Accession codes, unique identifiers, or web links for publicly available datasets
- A list of figures that have associated raw data
- A description of any restrictions on data availability

The P. protegens CHA0-GFP reference strain genome sequence, determined for this study, is deposited on NCBI GenBank: CP003190.1. The updated genome with full annotation is deposited on GenBank: RCSR00000000.1. Raw sequencing data used in this study are deposited at the NCBI database under BioProject PRJNA473919. Raw data of P. protegens CHA0 phenotypic traits, Supplementary dataset 1 and 2, are deposited at Mendeley Data: doi: [10.17632/3g6db3pj6b.1]. Used r-code is deposited in Github with DOI: https://doi.org/10.5281/zenodo.4750789.

Field-specific reporting

Please select the one below that is the best fit for your research. If you are not sure, read the appropriate sections before making your selection.

☐ Life sciences ☐ Behavioural & social sciences ☒ Ecological, evolutionary & environmental sciences

For a reference copy of the document with all sections, see nature.com/documents/nr-reporting-summary-flat.pdf

Ecological, evolutionary & environmental sciences study design

All studies must disclose on these points even when the disclosure is negative.

Study description	This study is based on five experimental evolutionary lines of P. protegens CHA0 evolving in the rhizosphere of the model plant Arabidopsis thaliana Col-0 (otherwise gnotobiotic study system). At each growth cycle (4 weeks), bacteria were harvested and kept for further analysis (for phenotyping and genome sequencing) as well as used for reintroduction to a fresh set of plants.
Research sample	Five replicated, independent evolutionary lines of bacteria. Six data collection time points
Sampling strategy	After each growth cycle, root-associated bacteria were harvested by placing the roots into a 1.5 ml Eppendorf tubes filled with 1 ml 10 mM MgSO4 and two glass beads. Rhizosphere bacteria were suspended into the liquid using a TissueLyser II at a frequency of 20 s-1 for 1 min after which bacterial cell densities were determined using flow cytometry (BD Accuri™ C6 Plus, thresholds for FSC: 2000, SSC: 8000). After this, 10^6 cells were inoculated to the rhizosphere of new A. thaliana plants to initiate the next plant growth cycle.<br>To quantify changes in plant-bacterium interaction, sixteen evolved bacterial colonies are randomly selected from each plant replicate selection line at the end of the second, fourth and sixth growth cycles, in addition to sixteen randomly selected ancestral colonies (in total 256 isolates). Sixteen evolved bacterial colonies is a reasonable sampling size to represent a population considering the practical posibility.
Data collection	Bacterial abundance data was collected by Erqin Li with flow cytometry (BD Accuri™ C6 Plus, thresholds for FSC: 2000, SSC: 8000). Phenotypic data were collected manually in specific bioassays by Chen Liu and Erqin Li with spectrophotometer (SPECTROstar Nano). Plant-bacterium interaction data were collected by Erqin Li and Chen Liu with balance and ImageJ (version 1.50i). Genomic DNA was sequenced using standard procedures by Erqin Li. Bacterial relative fitness data was collected by Henan Jiang and Erqin Li with LightScanner (Idaho Technology Inc.)
Timing and spatial scale	The selection experiment started on 2015. 01, and ended on 2016. 05. The phenotypic data, interaction data were collected from 2016. 05-2017. 12. Bacterial genome sequencing library preparation were done during 2016.11-2016.12. Sequencing data analysis was conducted during 2017. 05-2018. 05. Bacterial relative fitness measurements were conducted between 2018.05 to 2019.03<br>There was no spatial scale; plants were regularly randomized in plant growth chamber.
Data exclusions	All data were kept in the statistical analysis
Reproducibility	The experiment was based on five fully independent replicate lines.
Randomization	Plants were randomized every second day to prevent block/ position effect inside plant growth chamber.
Blinding	Data from phenotypic assays, bacterial abundance, plant-microbe interaction, as well as sequencing data were collected as a series of numbered samples, with a file listing which belonged to which treatment.

Did the study involve field work? ☐ Yes ☒ No

Reporting for specific materials, systems and methods

We require information from authors about some types of materials, experimental systems and methods used in many studies. Here, indicate whether each material, system or method listed is relevant to your study. If you are not sure if a list item applies to your research, read the appropriate section before selecting a response.

Materials & experimental systems

n/a	Involved in the study	
☒	☐	Antibodies
☒	☐	Eukaryotic cell lines
☒	☐	Palaeontology and archaeology
☒	☐	Animals and other organisms
☒	☐	Human research participants
☒	☐	Clinical data
☒	☐	Dual use research of concern

Methods

n/a	Involved in the study	
☒	☐	ChIP-seq
☐	☒	Flow cytometry
☒	☐	MRI-based neuroimaging

Flow Cytometry

Plots

Confirm that:

- [x] The axis labels state the marker and fluorochrome used (e.g. CD4-FITC).
- [x] The axis scales are clearly visible. Include numbers along axes only for bottom left plot of group (a 'group' is an analysis of identical markers).
- [x] All plots are contour plots with outliers or pseudocolor plots.
- [x] A numerical value for number of cells or percentage (with statistics) is provided.

Methodology

Sample preparation	Bacterial cell suspension were harvested from roots into buffer 10 mM MgSO4, and processed by flow cytometry after 20 times (10ul into 190ul) dilution.
Instrument	Accuri C6
Software	Accuri C6
Cell population abundance	Root suspension was directly processed in the machine. GFP signal allowed for separating the bacteria from root debris.
Gating strategy	Cells gated based on their SSC and FL1 (GFP fluorescence signal) to separate them from root debris and noise.

- [x] Tick this box to confirm that a figure exemplifying the gating strategy is provided in the Supplementary Information.
